# Supplementary figures and images for: Evidence for a non-linear carbon accumulation pattern along an Alpine glacier retreat chronosequence in Northern Italy
Source: PeerJ. 2019 Oct 10;7:e7703. doi: 10.7717/peerj.7703 (PMC6790226; doi:10.7717/peerj.7703)

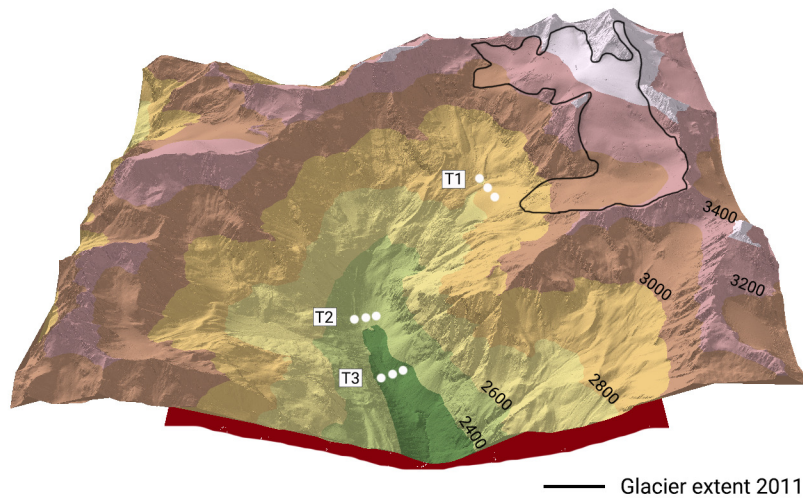

Supplement: Supplemental Information 1 — T1, T2 and T3 indicate the experimental transects. The continuous black contour line indicates glacier extent in 2011. [file peerj-07-7703-s001.pdf]
