# Supplementary material for: Evidence for a non-linear carbon accumulation pattern along an Alpine glacier retreat chronosequence in Northern Italy
Source: PeerJ. 2019 Oct 10;7:e7703. doi: 10.7717/peerj.7703 (PMC6790226; doi:10.7717/peerj.7703)
Supplement: Supplemental Information 2 — The letter T indicates the transect (T1–T3) and the letter C indicates the collar (C1–C5). [file peerj-07-7703-s002.pdf]

S2_Images of the collars and the sampled portions of ecosystem.

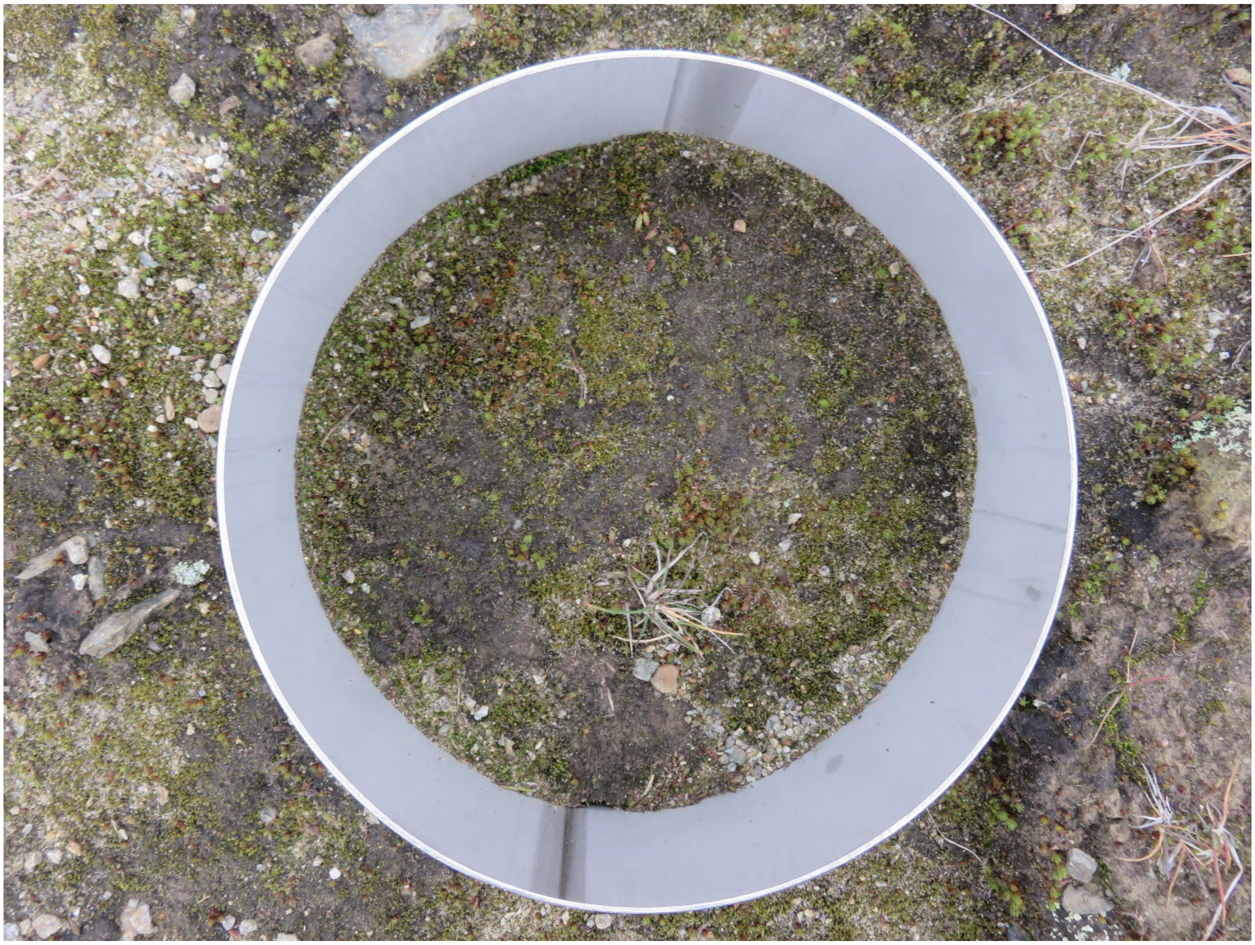

T1C1

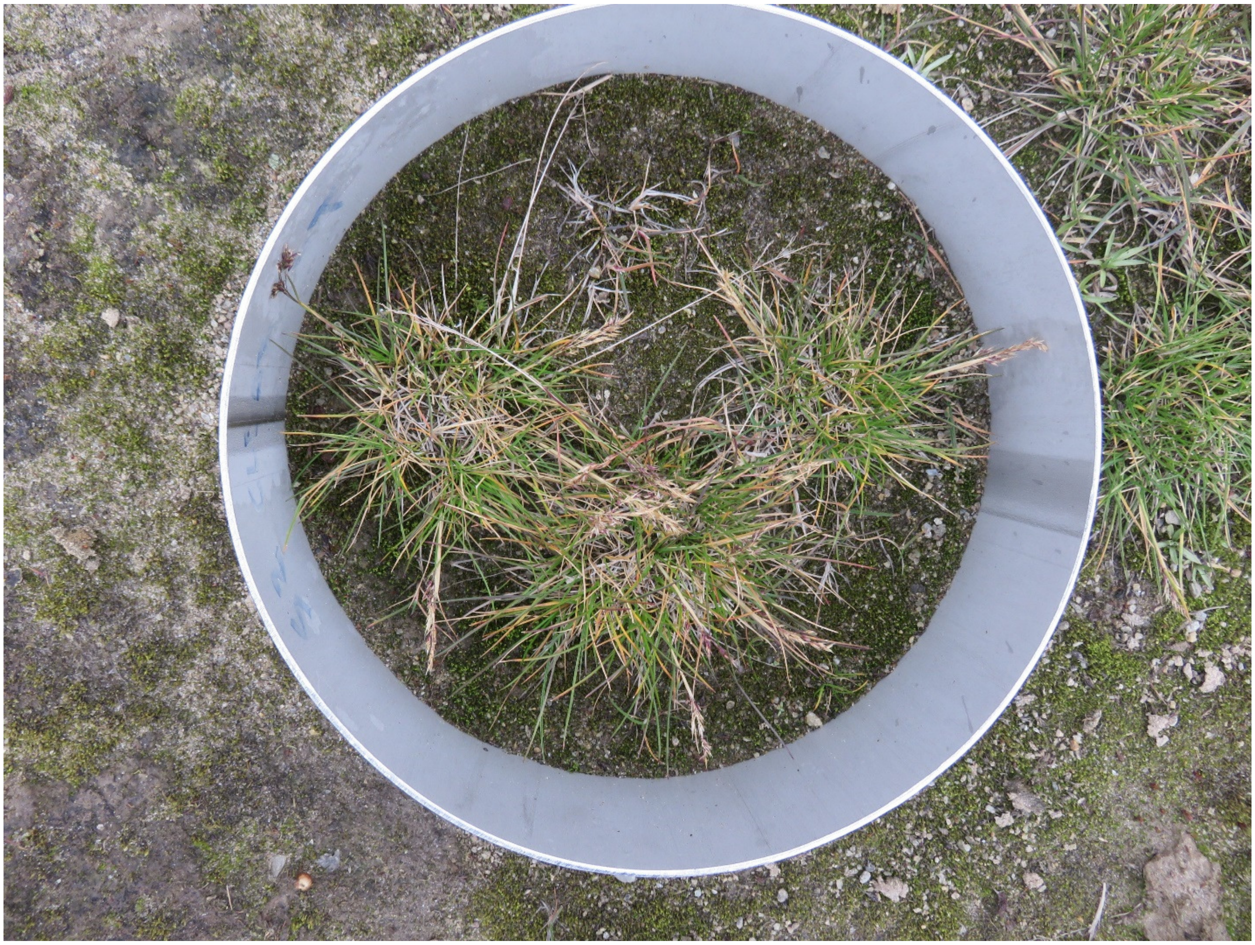

T1C2

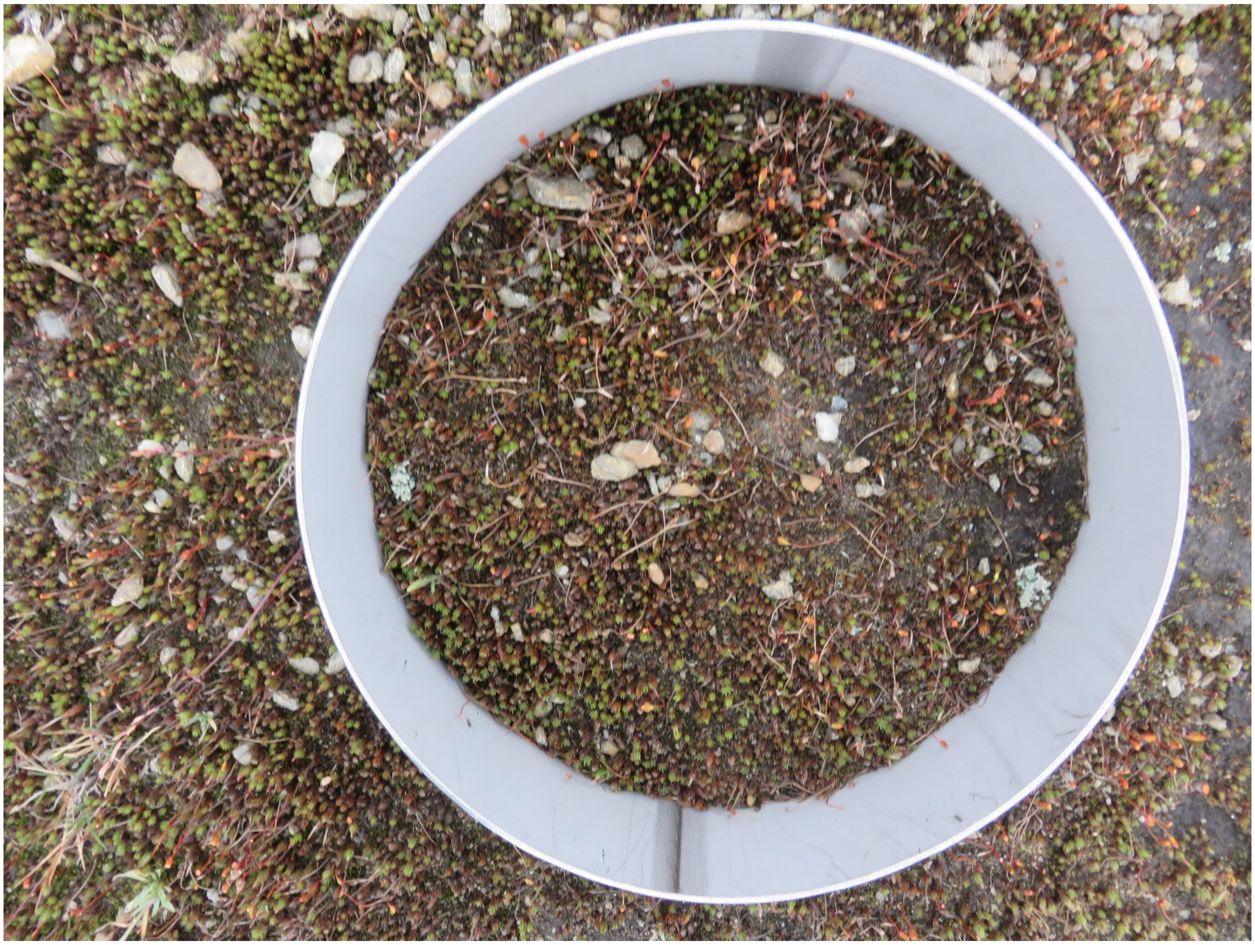

T1C3

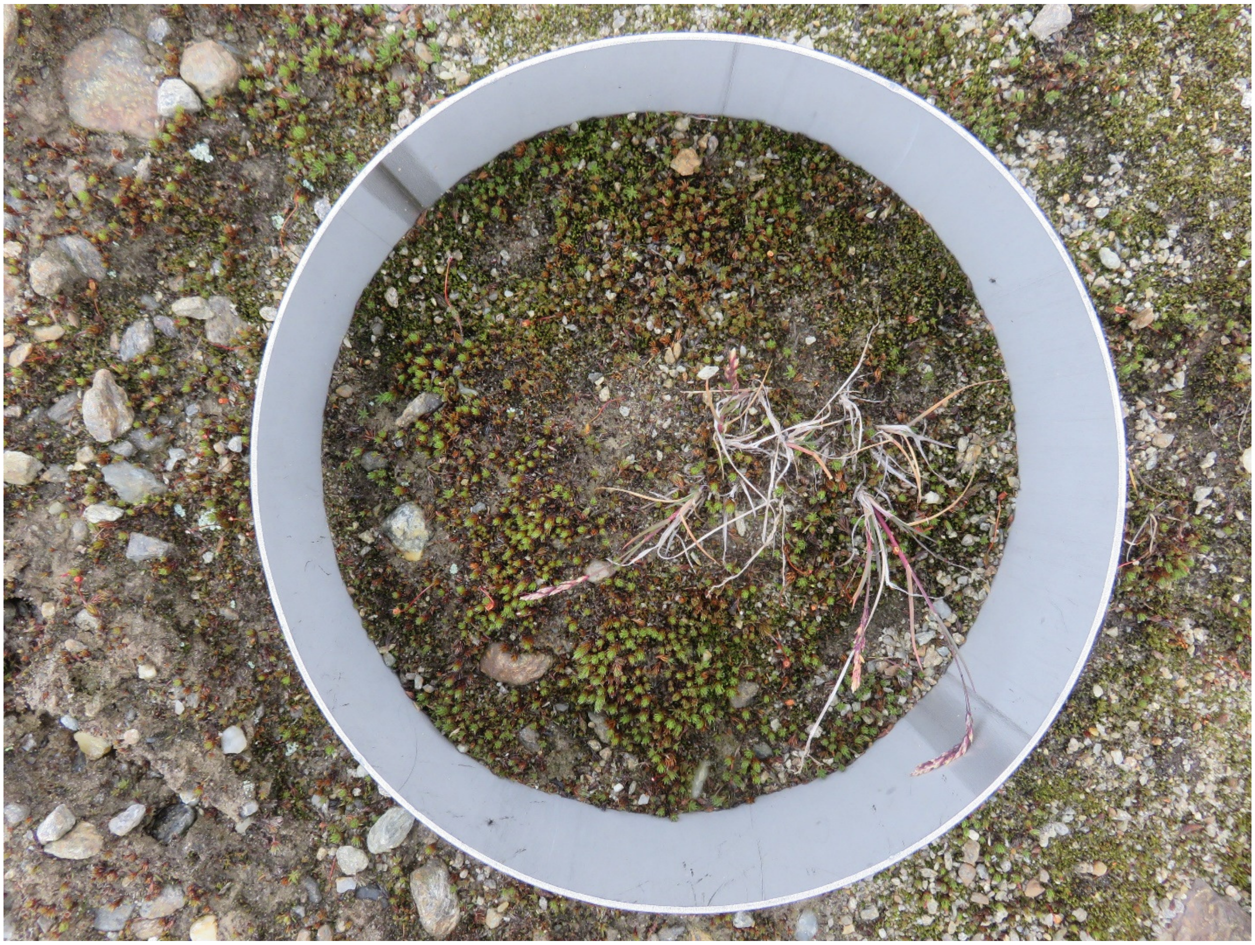
T1C4

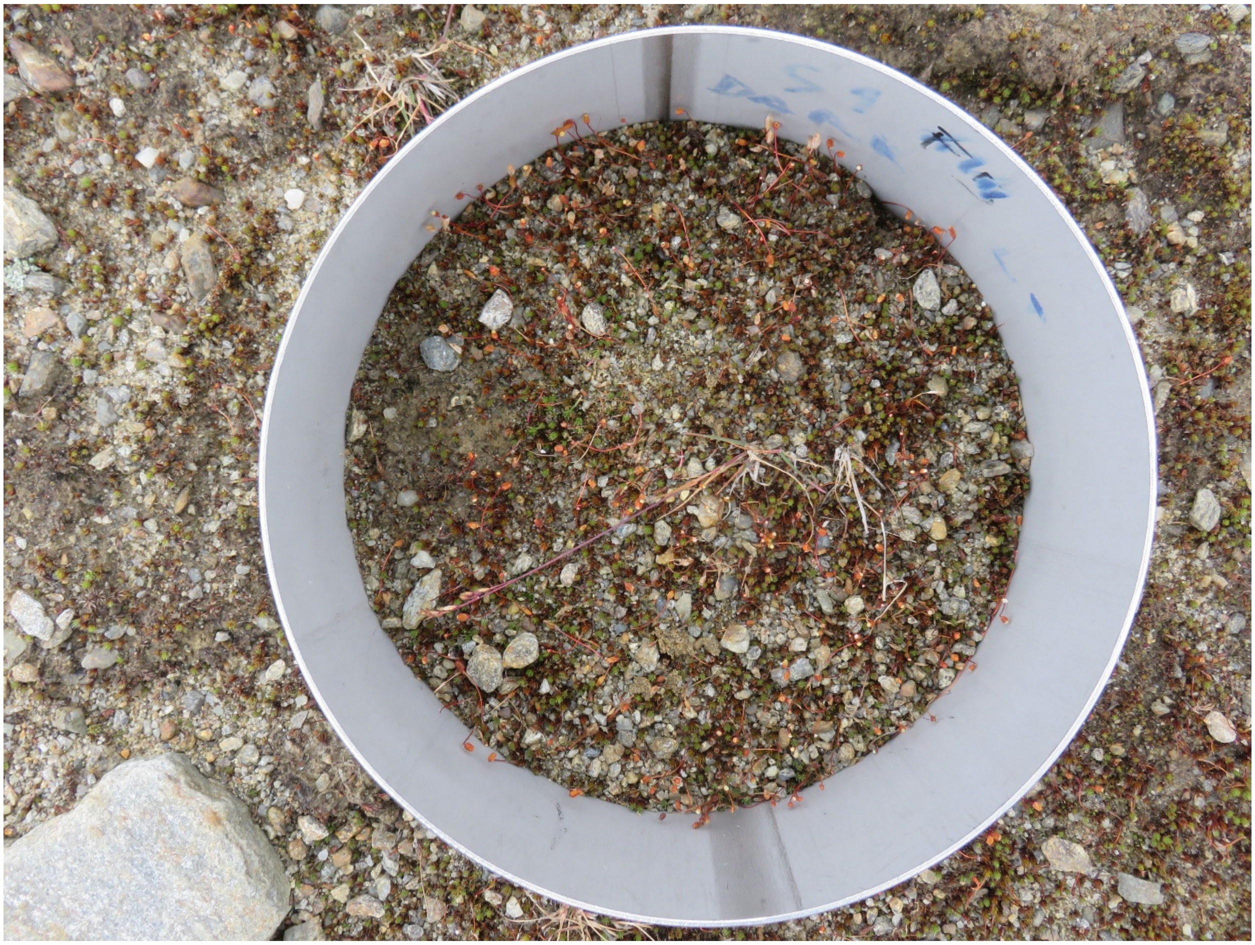

T1C5

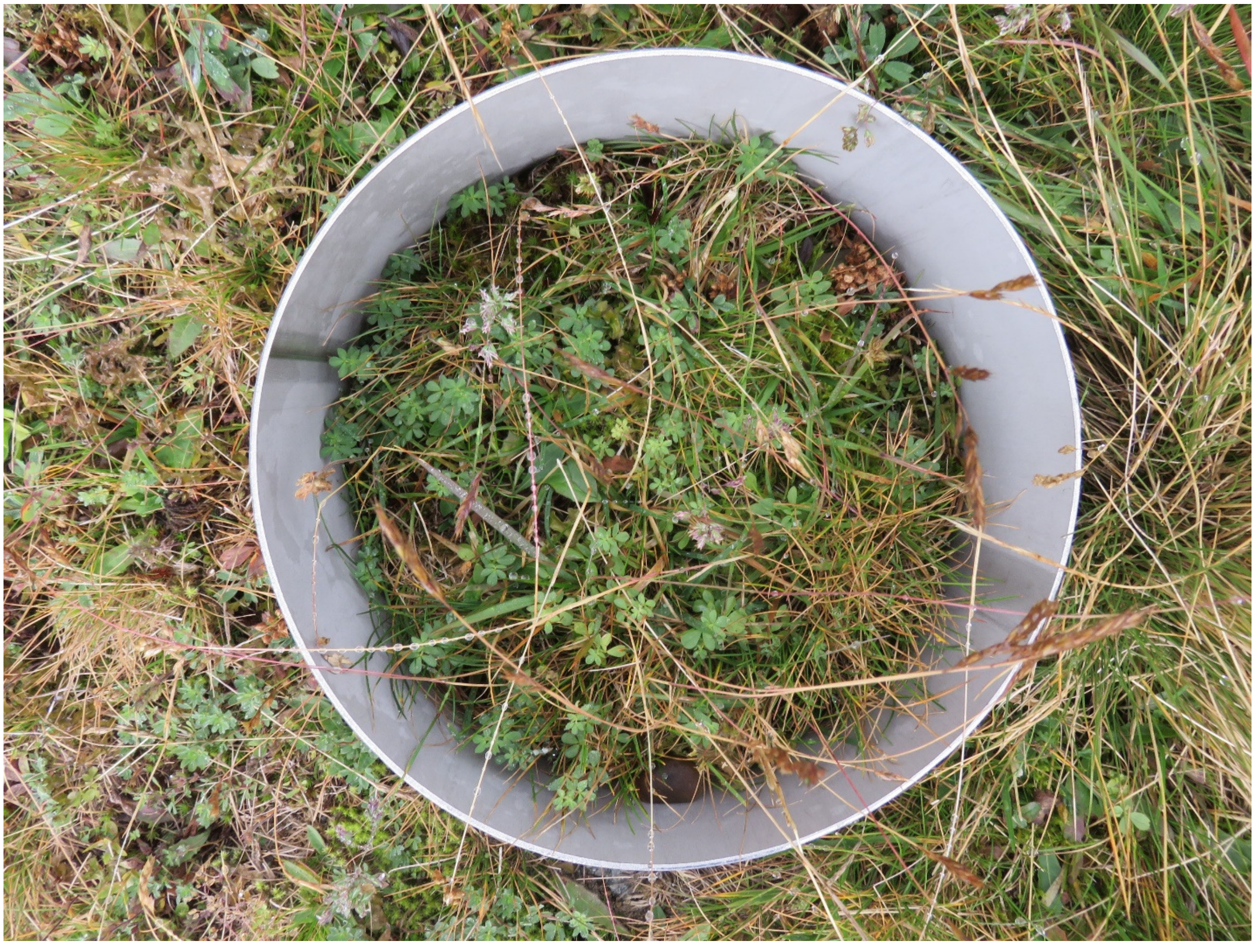

T2C1

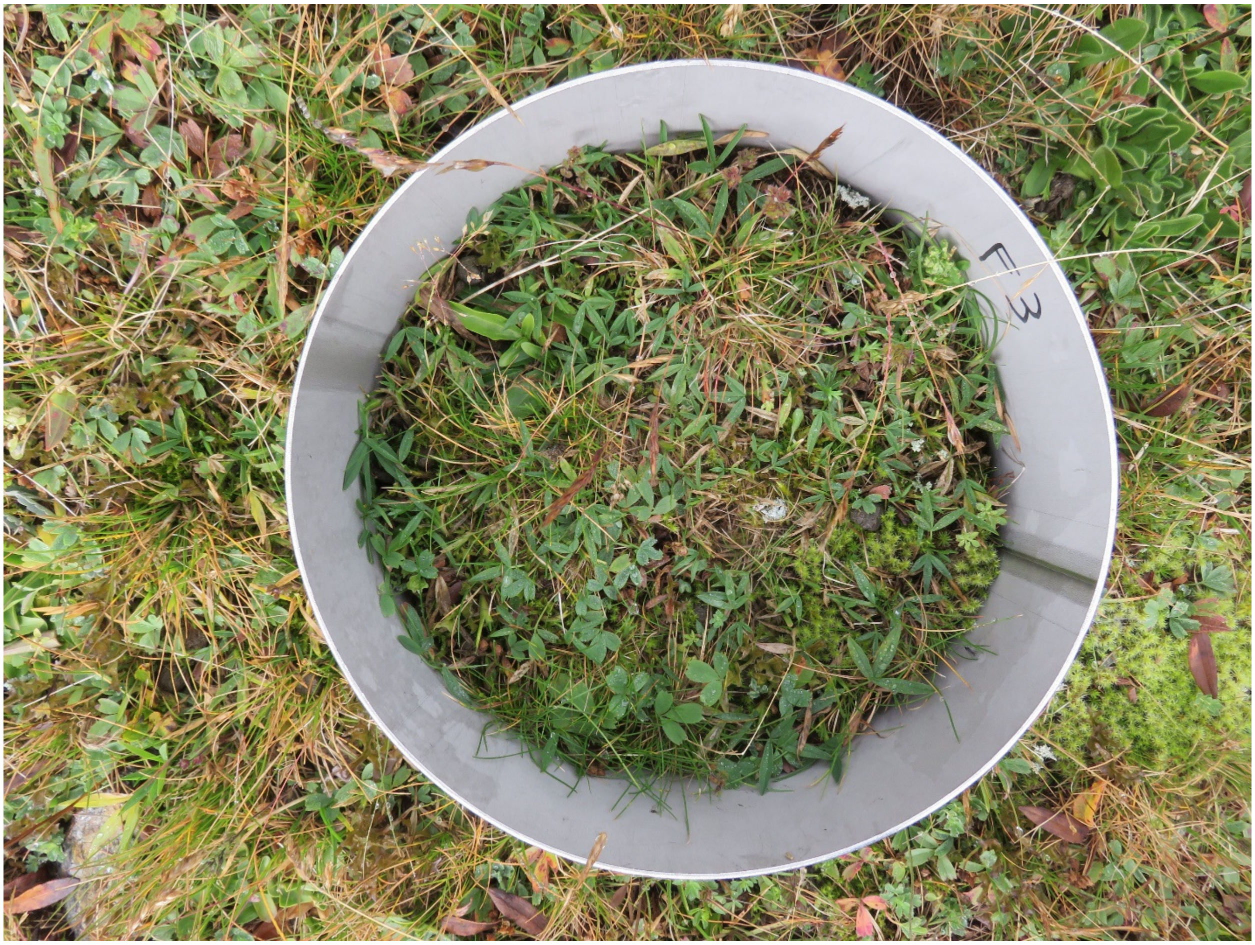

T2C2

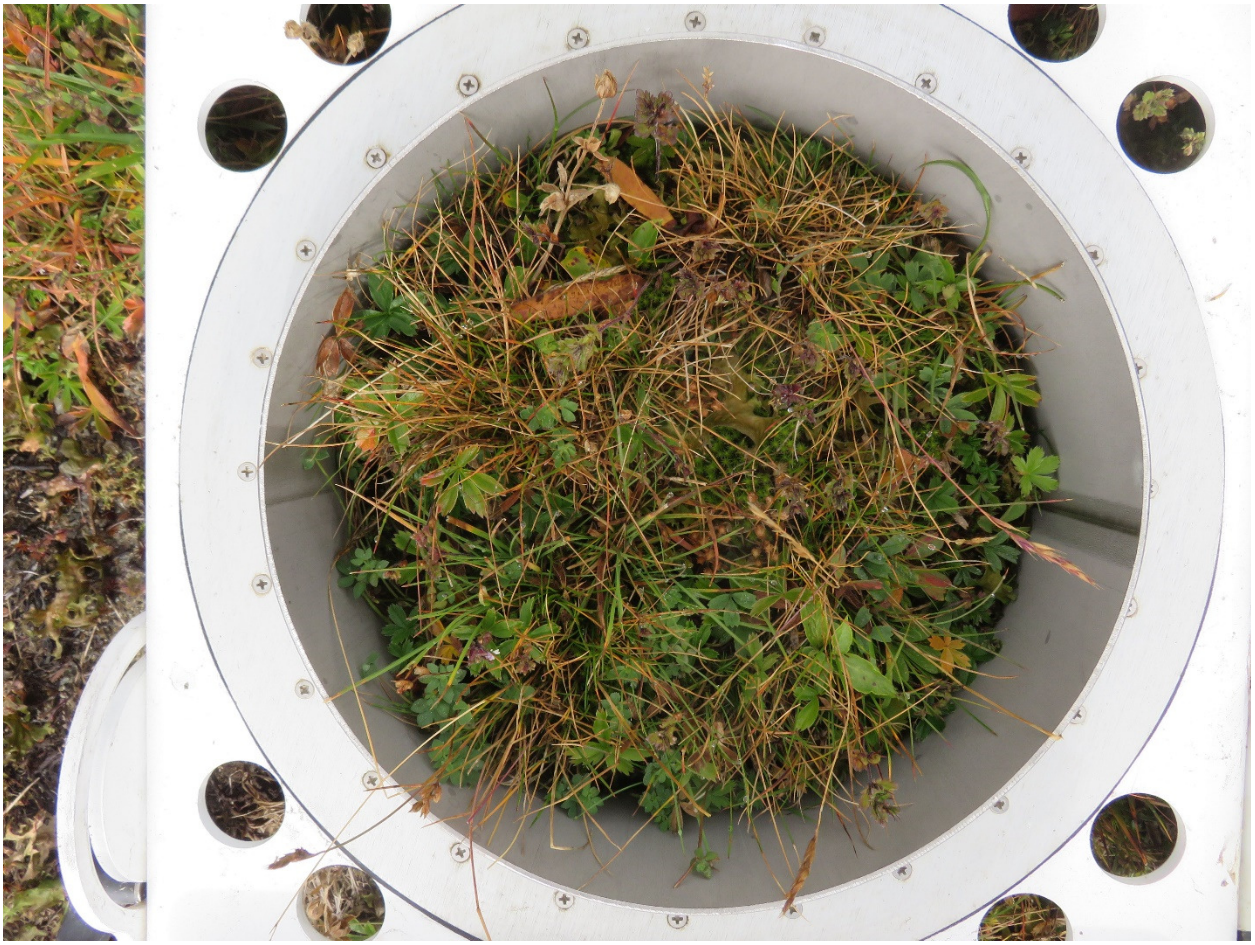

T2C3

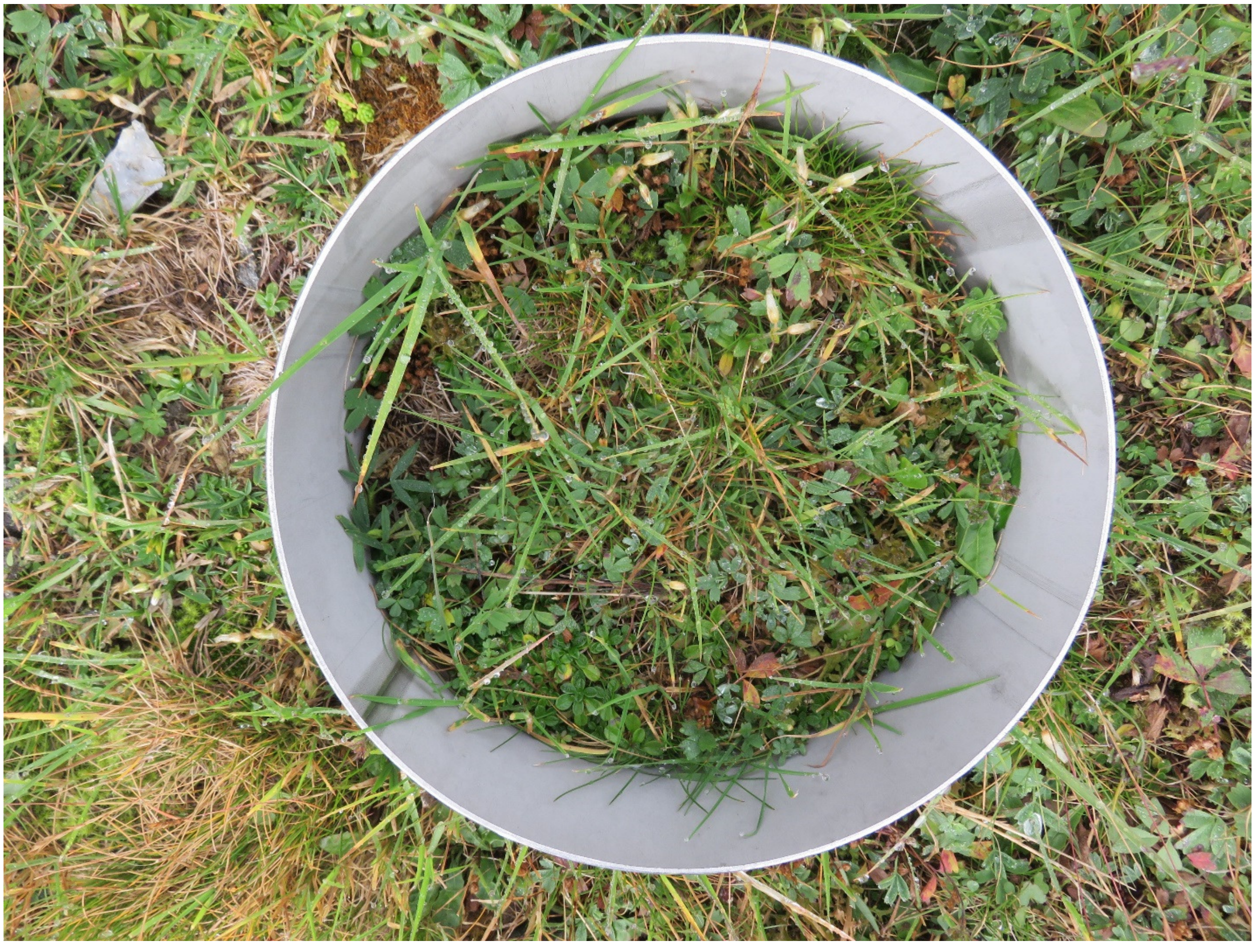

T2C4

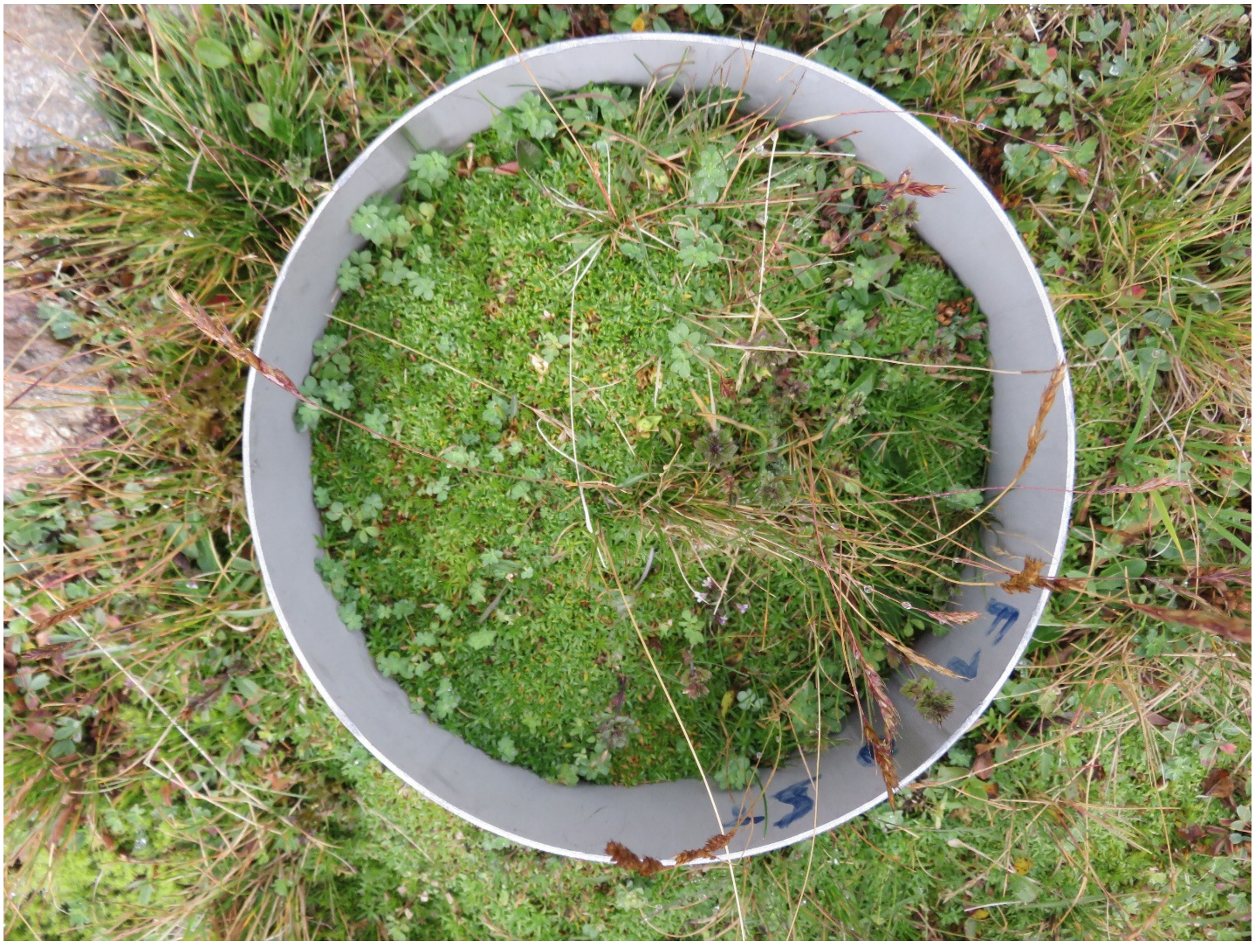

T2C5

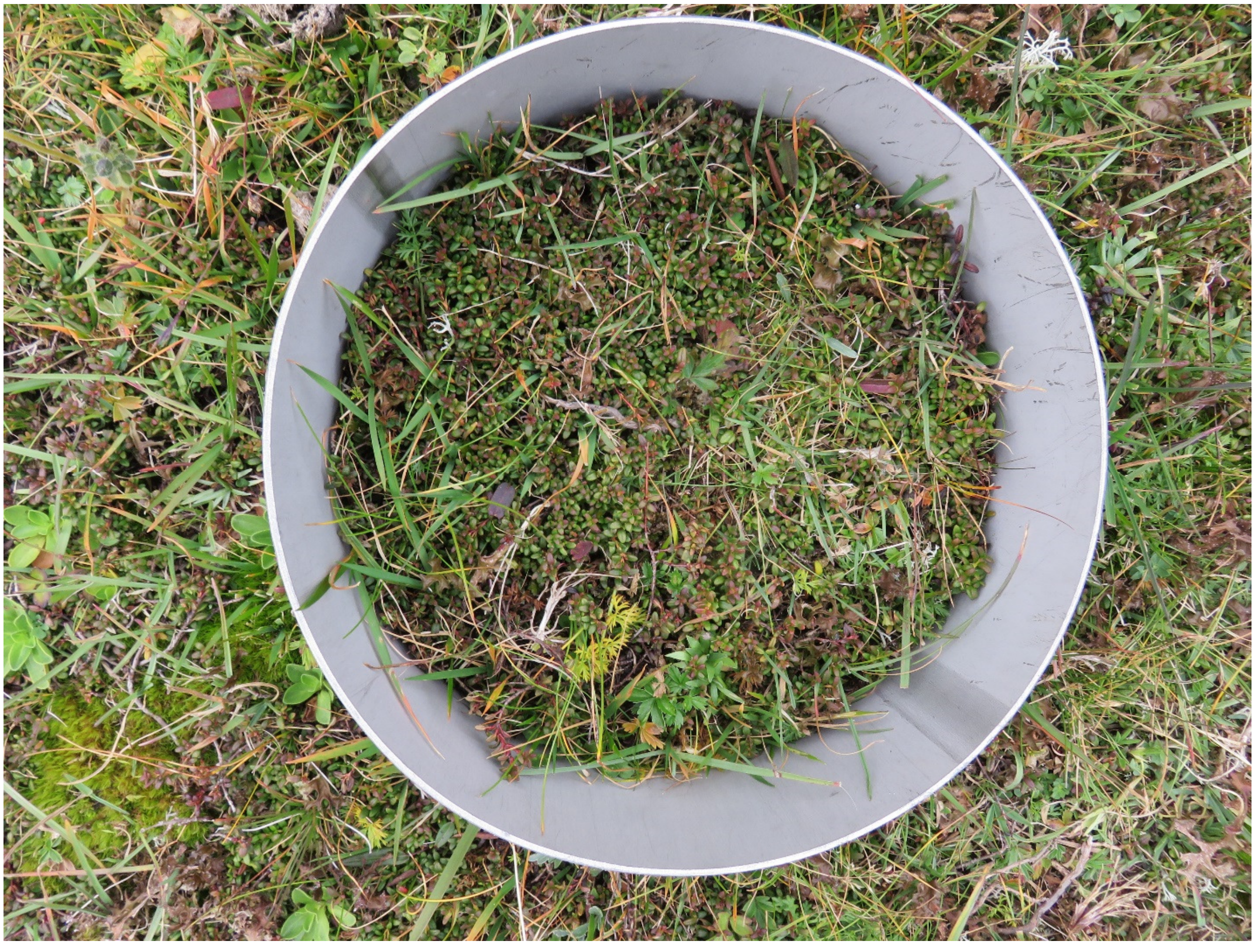

T3C1

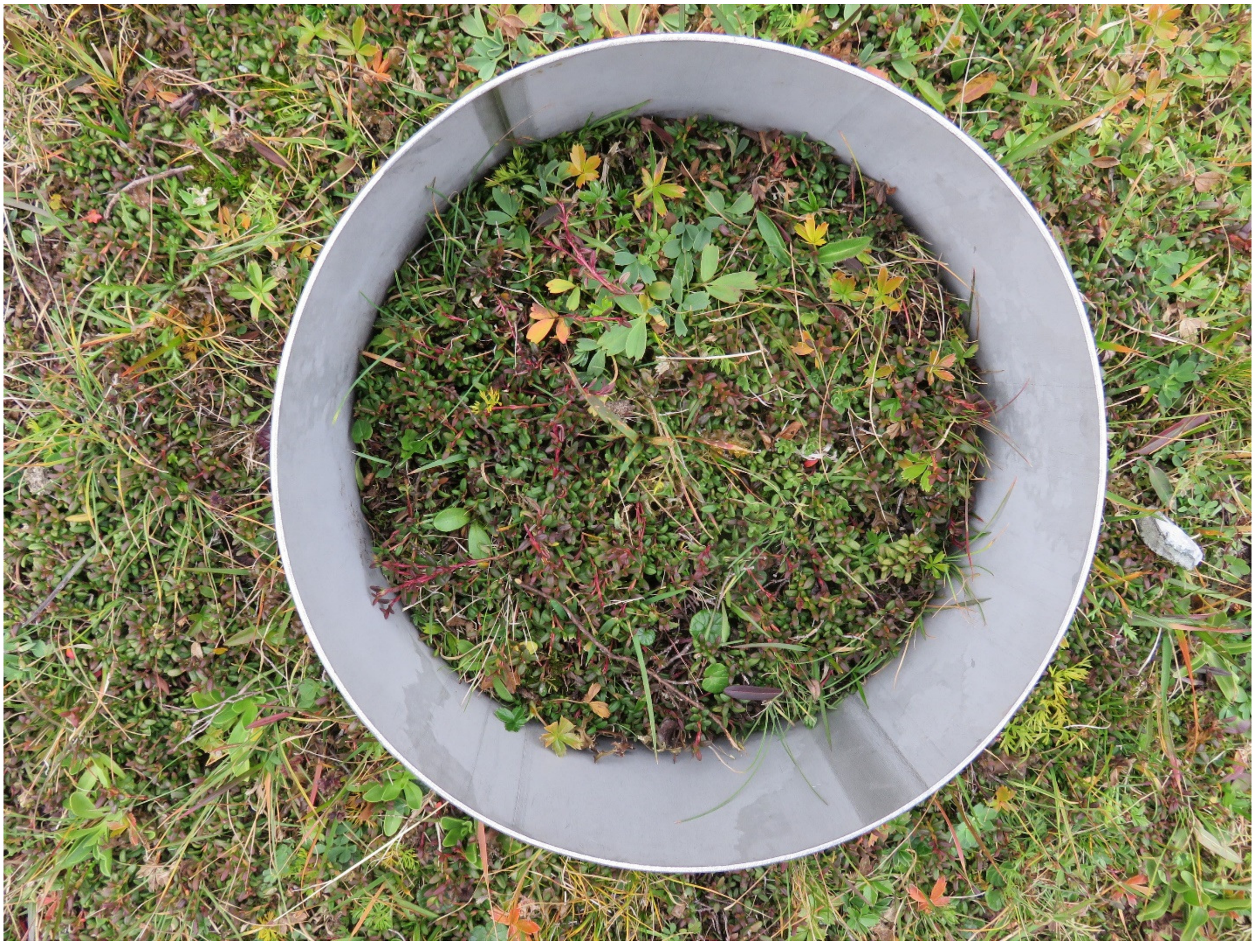

T3C2

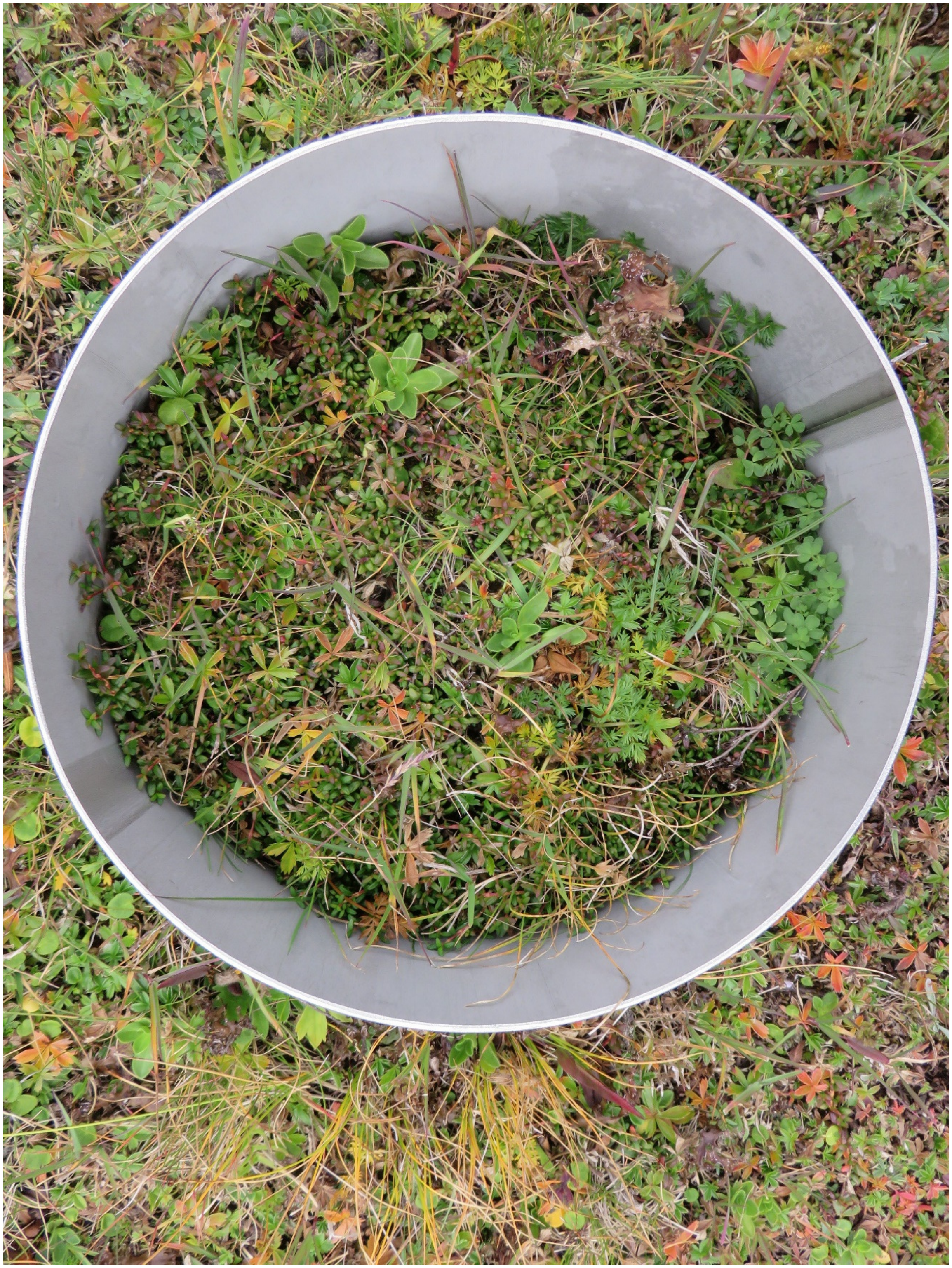

T3C3

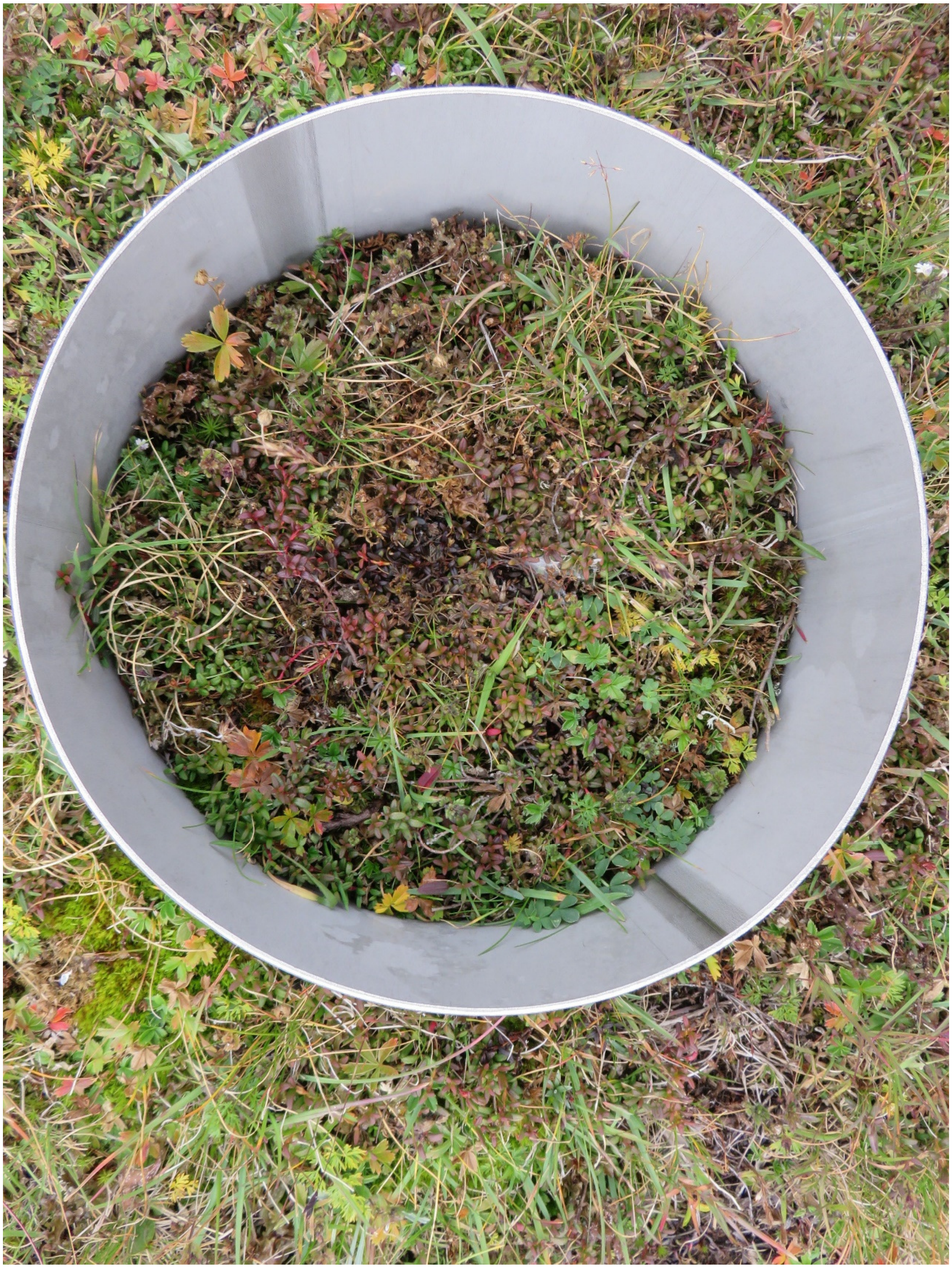

T3C4

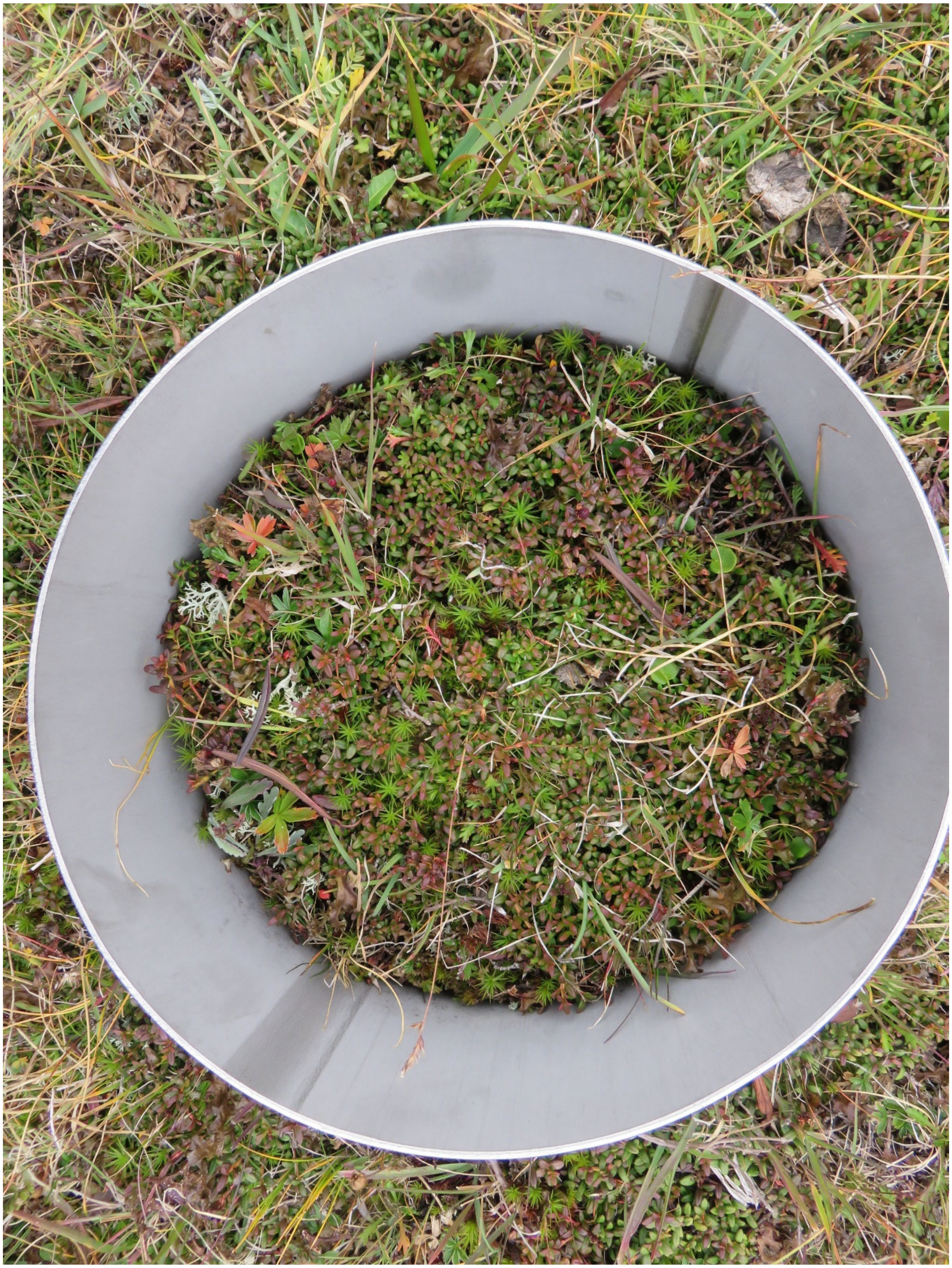

T3C5
